# Editorial: Advances in neurorehabilitation strategies for children with rare neurological disorders

**DOI:** 10.3389/fnhum.2024.1415096

**Published:** 2024-04-24

**Authors:** Cristiano M. Verrelli, Agata Polizzi, Martino Ruggieri, Fabio della Rossa, Marco Iosa

**Affiliations:** ^1^Department of Electronic Engineering, University of Rome Tor Vergata, Rome, Italy; ^2^Department of Educational Science, Pediatrics, University of Catania, Catania, Italy; ^3^Unit of Pediatric Clinic, Department of Clinical and Experimental Medicine, University of Catania, Catania, Italy; ^4^Department of Electronic, Information and Biomedical Engineering, Politecnico di Milano, Milan, Italy; ^5^Department of Psychology, Faculty of Medicine and Psychology, Sapienza University of Rome, Rome, Italy; ^6^Santa Lucia Foundation (IRCCS), Rome, Italy

**Keywords:** rare disease, modified constraint-induced movement therapy, motor imagery, instrument movement analysis, antispasmodic rehabilitation

Rare diseases [**RDs**] are usually multisystemic conditions affecting < 5 in 10,000 individuals in the general population. Despite the rarity of each single disorder, taken all together they affect over 300 million individuals worldwide, and more than 50% of these people are children with different phenotypes and onsets. Nearly 80% of these subjects have neurological manifestations causing life-long motor, intellectual and psychosocial disabilities of various types and severity. The main neurological RDs include nervous system malformations, disorders of neurodevelopment, epileptic encephalopathies, neurometabolic and neuromuscular diseases, mitochondrial and neurotransmitter disorders, movement disorders including ataxias and spastic paraplegias, immune-mediated neurological disorders, DNA defect repair syndromes, and neurocutaneous syndromes. Affected people with neurological RDs experience multiple accesses to different health services and multifaceted, often not conclusive treatments. Among a few conditions which can be currently cured, care is entrusted to forms of tailored management and symptomatic medications, coupled with programs of physical/neurocognitive rehabilitation. The time spent by these children in rehabilitation affects school attendance and performance, study time, playful hours, and leisure activities; this is aggravated in adolescents and adults, including parents, by the absence from employment and job productivity. Nevertheless, in many low/middle-income countries more than half of people with RDs do not receive the rehabilitation services they require. The need for continuity of special care encourages researchers to put in action new strategies, combining innovative approaches for motor and cognitive learning based on new approaches and emerging technologies.

The present Research Topic is part of this vibrant and dynamic context. It consists of 4 research studies coming from different research fields including bio-engineering, habilitation/rehabilitation, general medicine, pediatrics, psychology, and educational sciences, thus reflecting the innovative results and tools of multidisciplinary approaches, which are typical of complex systems and analyses including the use of modified constraint-induced movement therapy, instrument movement analysis, antispasmodic rehabilitation coupled with drug therapy and motor imagery.

⋆ Cui et al. suggest the use of a *modified Constraint-Induced Movement Therapy* [m**CIMT**] in children with consequences of unilateral brachial plexus injury, where CIMT consisted in splinting the unaffected arm to induce the movements of the affected counterpart, avoiding the learned non-use, while being mainly used in adults with stroke (Smania et al., 2012). A randomized controlled trial is conducted on 36 children: both groups undergo physiotherapy intervention. The protocol for the experimental group implies the application of mCIMT for 4 hours per day over 90 days. At baseline, as well as at the end of the intervention, no statistically significant differences are noted; however at the 6-month follow-up time point, the mCIMT group shows significant differences in terms of scores [in favor of the mCIMT group] in the Active Movement Scale, Mallet Shoulder Scale, and Gilbert-Raimondi Elbow Scale.
⋆ Layne et al. propose the use of *instrument movement analysis* for assessing walking abilities in a 9-year-old child affected by SYNGAP1 [Synaptic Ras GTPase-Activating Protein 1] syndrome, a rare neurodevelopmental condition characterized by gross motor delays, profound intellectual disability, and behavioral deficits. In this study, the control subject is the proband neurotypical fraternal female twin. Many kinematic significant differences emerge in terms of: **(**i) symmetry of movements; **(**ii) joint range of motion; **(**iii) angular velocities of specific anatomical landmarks. This instrumented, quantitative, and hence objective approach might be helpful for assessing the development of locomotor abilities, and hence the efficacy of therapeutic interventions.
⋆ Chen et al. report the case of three individuals within the same family affected by spinocerebellar ataxia type 8 caused by *ATXN8OS* gene variants with spasticity onset in early childhood. Manifestations of the disease include spastic dyskinesia and cerebellar atrophy. The authors show that the combination of *systematic antispasmodic rehabilitation* coupled with the use of oral drugs, botulinum toxin injection and baclofen pump, improves the daily life of these persons.
⋆ Gentile et al. investigate the innovative approach of *motor imagery* [**MI**]. According to the functional equivalence theory, the imagination of a movement has many aspects in common with the actual execution of that movement, especially those regarding the activation of the same motor areas (Jeannerod, 1994). The use of this property in clinical contexts was investigated prevalently in adults with neurological disorders. The systematic review of the literature by Gentile et al. identifies 22 original studies involving 476 children (aged 5–18 years) with 10 different neurological conditions including, cerebral palsy spectrum disorders, stroke, coordination disorders, intellectual disabilities, brain and/or spinal cord injuries, autism spectrum disorders, pain syndromes, and hyperactivity. Nineteen out of 22 studies claim for the efficacy of MI. In the light of these results, the authors suggest that MI could be a reliable supportive/add-on (home-based) rehabilitative tool for pediatric neurorehabilitation. However, its clinical use in children is highly dependent on the complexity of MI mechanisms that are related to the underlying neurodevelopmental disorder.

Certainly, the great variability of different disorders and multiplicity of underlying pathogenic mechanisms involved and analyzed in the systematic review, in the case series and in the case reports included in this Research Topic, and more in general reported in the current literature, highlight the caveats and limits of research on neurorehabilitation strategies in rare neurological disorders, especially in children. International research collaborations, projects as well as Research Topics such those included in this Research Topic, can be thus helpful for highlighting and providing an in-depth understanding of the neurological manifestations of RDs and of the efficacy of (re)-habilitation interventions. while being able to stimulate the use of such insights in the clinical practice.

## Author contributions

CV: Writing – original draft, Writing – review & editing. AP: Writing – original draft, Writing – review & editing. MR: Writing – original draft, Writing – review & editing. FR: Writing – review & editing. MI: Writing – original draft, Writing – review & editing.
